# Correlation between negative expression of pepsinogen C and a series of phenotypic markers of gastric cancer in different gastric diseases

**DOI:** 10.1002/cam4.1615

**Published:** 2018-07-02

**Authors:** Jingyi Jiang, Shixuan Shen, Nannan Dong, Jingwei Liu, Qian Xu, Liping Sun, Yuan Yuan

**Affiliations:** ^1^ Tumor Etiology and Screening Department of Cancer Institute and General Surgery Liaoning Provincial Education Department The First Affiliated Hospital of China Medical University, and Key Laboratory of Cancer Etiology and Prevention (China Medical University) Shenyang China; ^2^ Department of Biochemistry & Molecular Biology China Medical University Shenyang China

**Keywords:** correlation, gastric disease, pepsinogen C, tumor marker

## Abstract

Gastric tumorigenesis is a multistep process initiated by chronic superficial gastritis (SG), followed by atrophic gastritis (AG), then intestinal metaplasia (IM), and finally by dysplasia and adenocarcinoma according to the Correa model. Pepsinogen C (PGC) decreases gradually during progression of cancer, which makes PGC an ideal negative marker for GC. To explore the correlation between PGC and other positive tumor markers in different gastric diseases, we observed the expression of PGC, MG7‐Ag, MMP9, NM23, Ki‐67, and E‐cadherin by immunohistochemistry, quantitative RT‐PCR, and immunoblot analysis. Our results showed that in SG, PGC was highly expressed while malignant phenotype markers were rarely expressed. In contrast with SG, malignant phenotype markers were highly expressed while the positive rate of PGC reached only 1.44% in GC. So there was no coexpression of PGC and malignant phenotype markers in SG or GC tissues. Only in the AG group, which is well‐known to be gastric precancerous disease, coexpression of PGC and malignant phenotype markers was detected. Our results suggested that the expression of PGC in AG was negatively correlated with that of MG7‐Ag and MMP9. Of all AG, those with low expression of PGC and high expression of MG7‐Ag and MMP9 may possess a greater potential of malignant transformation. Combined detection of negative marker PGC and positive markers MG7‐Ag and MMP9 could be used as a potential follow‐up panel for monitoring dynamical progression of AG and improving the detection efficiency of high‐risk individuals of gastric cancer, and then taking necessary interventions on the target population.

## 1 INTRODUCTION

As is known to all, tumorigenesis is a multistep process with various factors involved.^1^ The transformation of normal cells into tumor cells involves a series of biological changes. The initiation of cancer is extremely complicated, which includes aberrant differentiation, uncontrolled proliferation, resisting apoptosis, activating invasion and metastasis, and inducing angiogenesis.^2^ As these unique biological behaviors are mediated by intricate molecular pathways, any component may be tumor molecular imprinting and constitute the phenotypic characteristics of tumor. The phenotypic characteristics of tumor can be explored in different stages of tumorigenesis from presence and absence (increase/decrease) aspects. This can be of benefit to our understanding of histogenesis, cell differentiation, and dysfunction of tumor, but also contribute to prediction, diagnosis, classification, prognosis, and treatment of cancer.

Gastric cancer (GC) remains the fifth most common malignancy and the third leading cause of cancer death in both sexes worldwide.^3^ Likewise, carcinogenesis of GC is a multistep process initiated by chronic superficial gastritis (nonatrophic gastritis, SG), followed by atrophic gastritis (AG), then intestinal metaplasia (IM), and finally by dysplasia and adenocarcinoma.^4^, ^5^ Atrophic gastritis (clinical terminology), intestinal metaplasia, and dysplasia (histopathological terminology) are well‐established premalignant conditions of GC.^6^, ^7^, ^8^ Effective management of AG, IM, and dysplasia is a very important way to prevent GC. Current studies have shown that the incidence rate of progression from GA, IM, and dysplasia to GC varies widely by geographical location.^9^ What is more, not all patients with premalignant conditions of GC will develop cancer. So further assessment of this risk status should be performed to identify individuals who are most likely to become cancerous. In addition, based on logical thinking, some molecular markers with the potential of reflecting the malignancy risk in premalignant conditions of GC may contribute to identifications of individuals at high risk of GC.

Pepsinogen C (PGC) is the precursor of pepsin C, which belongs to the aspartic proteinase family.^10^ PGC is mainly synthesized by gastric chief cells and secreted into gastric lumen where it can be activated to digest protein from food.^11^ Several studies have suggested that PGC is highly expressed in normal gastric tissue, while rarely expressed in GC tissues.^12^ Our previous results also indicated that the expression level of PGC gradually declined during SG‐AG‐GC progression.^13^ Melle et al^14^ also found that PGC was obviously reduced in gastric cancer tissue by ProteinChip Arrays and SELDI‐TOF MS. As a negative marker, PGC has demonstrated important value in the screening, diagnosis, and prognosis of gastric cancer.^15^, ^16^

On the other hand, in the development of GC, some abnormally expressed tumor markers can indirectly reveal proliferation, apoptosis, differentiation, and metastasis potential of tumor cells. In the current study, we evaluated the expression levels of PGC and a series of tumor markers such as monoclonal gastric cancer 7 antigen (MG7‐Ag), matrix metalloproteinase 9 (MMP9), NM23 nucleoside diphosphate kinase 1 (NM23), antigen identified by monoclonal antibody Ki‐67 (Ki‐67), and epithelial mesenchymal transition‐related protein E‐Cadherin in tissues from subjects with different gastric diseases. In addition, we explored the relationships of PGC with the above malignant phenotypic markers.

## 2 MATERIALS AND METHODS

### 2.1 Patients and tissue specimens

In this retrospective study, we enrolled 368 subjects with different gastric diseases, including SG, AG, and GC. Among them, 139 individuals with GC who had undergone gastrectomy were recruited from the Department of Surgical Oncology of the First Hospital of China Medical University between June, 2009 and May, 2011 (Details of characteristics of patients with GC are shown in Table S1). In addition, 126 subjects with SG and 103 subjects with AG had undergone gastroscopy biopsy at the Zhuanghe Gastric Diseases Screening Program in Liaoning Province, China during May, 2002 and June, 2009.^17^ Inclusion criteria for this study were the subjects diagnosed pathologically with SG, AG, and GC by 2 qualified pathologists according to the updated Sydney gastritis classification^18^ and WHO classification of tumors of digestive system.^19^ In our study, 126 individuals with mild SG were selected as a control group compared with atrophic gastritis (precancerous disease) and gastric cancer. Patients who had a history of other malignancies or received preoperative radiotherapy or chemotherapy were excluded from this study. This study project was authorized by the Ethics Committee of China Medical University, and the informed consent was signed by each subject. Related clinical data (sex, age, etc.) were extracted from questionnaire and medical records.

### 2.2 Immunohistochemistry

All tissue samples were processed in formalin fixation for 24 hours and then embedded in paraffin. Briefly 4‐μm slides were cut from paraffin tissue blocks, deparaffinized, and hydrated. Antigen retrieval was achieved by submerging the slides in citric acid buffer (pH 6.0; MVS‐0066; Maixin Inc., Fujian, China) for 90‐seconds at 100°C. Hydrogen peroxide and normal goat serum (component A and B, UltraSensitiveTM SP [Goat] IHC Kit 9719) were used for blocking endogenous peroxidase and nonreactive sites respectively. Primary antibodies were incubated for one hour at 25°C, and antibody binding was detected using the Streptavidin‐Peroxidase complex (component C and D, UltraSensitive™ SP [Goat] IHC Kit 9719) and diaminobenzidine tetrahydrochloride solution (DAB Kit‐1031; Maixin Inc.). Finally, the sections were counterstained with haematoxylin and then dehydrated and mounted. PBS buffer was substitute for primary antibodies as a negative control. PGC antibody (clone No. 2D5, 1:500 dilutions) was offered by Clinical Laboratory Institute of Japanese. In addition, MG7‐Ab (1:300 dilutions; initial concentration 2.7 mg/mL) was supplied by Institute of Digestive Diseases, Xijing Hospital, Fourth Military Medical University. The other antibodies ready‐to‐use without further dilution were purchased from Maixin Inc. (against MMP9, MAB‐0245; against NM23, RAB‐0105; against Ki‐67, RMA‐0542; against E‐cadherin, MAB‐0589).

### 2.3 Evaluation of immunohistochemistry

Two certified pathologists blindly evaluated immunostaining of every section. The intensity and prevalence was assigned respectively as described elsewhere.^20^ The comprehensive scoring was then determined by multiplying the intensity and prevalence score. The expression level was graded as: negative (−), score = 0; weak expression (+), score = 1‐4; moderate expression (++), score = 5‐8; and strong expression (+++), score = 9‐12.

### 2.4 RNA isolation and quantitative reverse transcriptase PCR

RNA from cells was extracted using standard Trizol (Invitrogen) protocol. 1 μg of total RNA was converted to cDNA using Prime Script™ RT Master Mix (Takara). Quantitative reverse transcriptase PCR were performed using SYBR^®^ Premix Ex Taq™ II (Takara) assays on an Mastercycler ep realplex (Eppendorf). Glyceraldehyde‐3‐phosphate dehydrogenase (GAPDH) was chosen to be endogenous controls. The 20 μL reaction system were composed of 10 μL 2× SYBR^®^ Premix Ex Taq™ II, 0.8 μL forward primer, 0.8 μL reverse primer, and 2 μL cDNA. Amplification conditions we adopted were 95°C for 2 minutes, followed by 35 cycles of 95°C for 15 seconds, 60°C for 30 seconds. All assays were conducted at least 3 times. Relative expression level of PGC mRNA was analyzed by 2−ΔΔCt.^21^ Specific primers for PGC and GAPDH were synthesized by Takara (Dalian, China) and are listed in Table S2.

### 2.5 Western blot analysis

Gastric cancer cells or frozen gastric cancer and adjacent tissues were lysed by RIPA lysis buffer (P0013C; Beyotime). Protein concentrations were quantified using the BCA protein assay reagent (P0009; Beyotime). 40 μg of protein for each sample were separated by 10% SDS‐PAGE and transferred electrophoretically to polyvinylidene fluoride membrane (Catalog No.88520; Thermo Scientific Pierce). The membranes were blocked in 5% skimmed milk prepared in TBS buffer with 1% Tween‐20 (TBST) at room temperature for one hour, and then incubated with primary antibodies overnight at 4°C. Subsequently the membranes were washed and then hybridized with secondary antibody for 2 hours at room temperature. After washing thoroughly with TBST, the membranes were detected using the ECL kit (Catalog No. 32106; Thermo Scientific Pierce) and protein bands on the membrane were scanned and analyzed with an imaging system (Tanon‐4200; Tanon Science & Technology Co., Ltd). The reagent for western blotting included PGC antibodies (ab9013; 1:3000 dilution in TBST; Abcam), MG7‐Ab (1:100 dilutions, initial concentration 2.7 mg/mL, supplied by Institute of Digestive Diseases, Xijing Hospital, Fourth Military Medical University), MMP9 antibodies (ab73734; 1:3000 dilution in TBST; 1:1000 dilution in TBST; Abcam), GAPDH antibodies (sc‐66163; 1:5000 dilution in TBST; Santa Cruz), horseradish peroxidase (HRP)‐conjugated anti‐goat or anti‐mouse secondary antibodies (ZB‐2306; or ZB‐2305; 1:1000 dilution in TBST; ZSGB‐BIO). Gray values of PGC/GAPDH were analyzed by Image J Statistical analysis.

### 2.6 Statistical analysis

SPSS (16.0) software (SPSS, Chicago, USA) was adopted for statistical analysis. The differences of immunostaining scores among different diseases were analyzed by Mann‐Whitney tests. The correlation of different protein was measured by Spearman's rank correlation coefficient test. The Chi‐square test or the Fisher's exact probability test was used to analyze the correlations between clinicopathological parameters of patients with GC and different protein. Wilcoxon Signed Ranks Test was used to analyze the differences of gray values of PGC/GAPDH between gastric cancer tissues and adjacent normal tissues. The error bars in histograms represent standard deviation (SD). All tests were 2‐tailed, and *P* < .05 was considered statistically significant.

## 3 RESULTS

### 3.1 Expression of PGC in different gastric diseases

**Figure 1:**
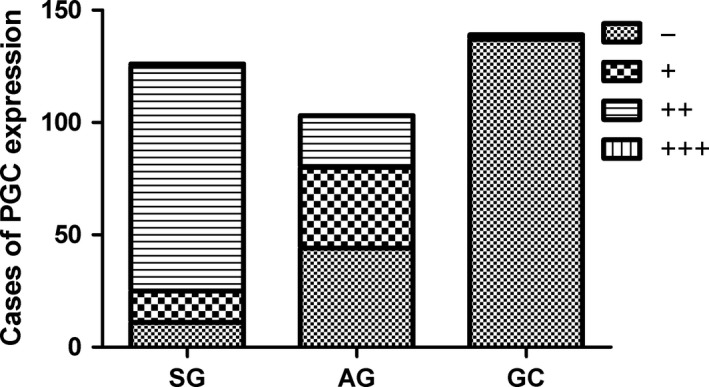
Expression of PGC in different gastric diseases by immunohistochemistry. −, negative; +, weak; ++, moderate; +++, strong staining

Our immunostaining results demonstrated that the positive expression of PGC in the SG, AG, and GC groups were 91.27%, 57.78%, and 1.44% respectively. Compared to the SG group, the expression of PGC significantly reduced in the AG and GC groups respectively (Figure 1, Figure S1). In addition, the expression of PGC was far lower in gastric cancer tissues than that of corresponding normal tissue by western blot (Figure S2).

### 3.2 Expression of malignant phenotype markers in different gastric diseases

**Figure 2:**
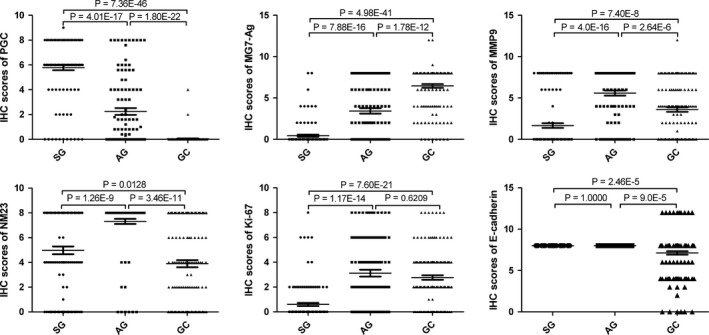
Expression of PGC, MG7‐Ag, MMP9, NM23, Ki‐67 and E‐cadherin in different gastric diseases. Mann‐Whitney tests was used to analyze the differences of immunostaining scores among different diseases

**Figure 3:**
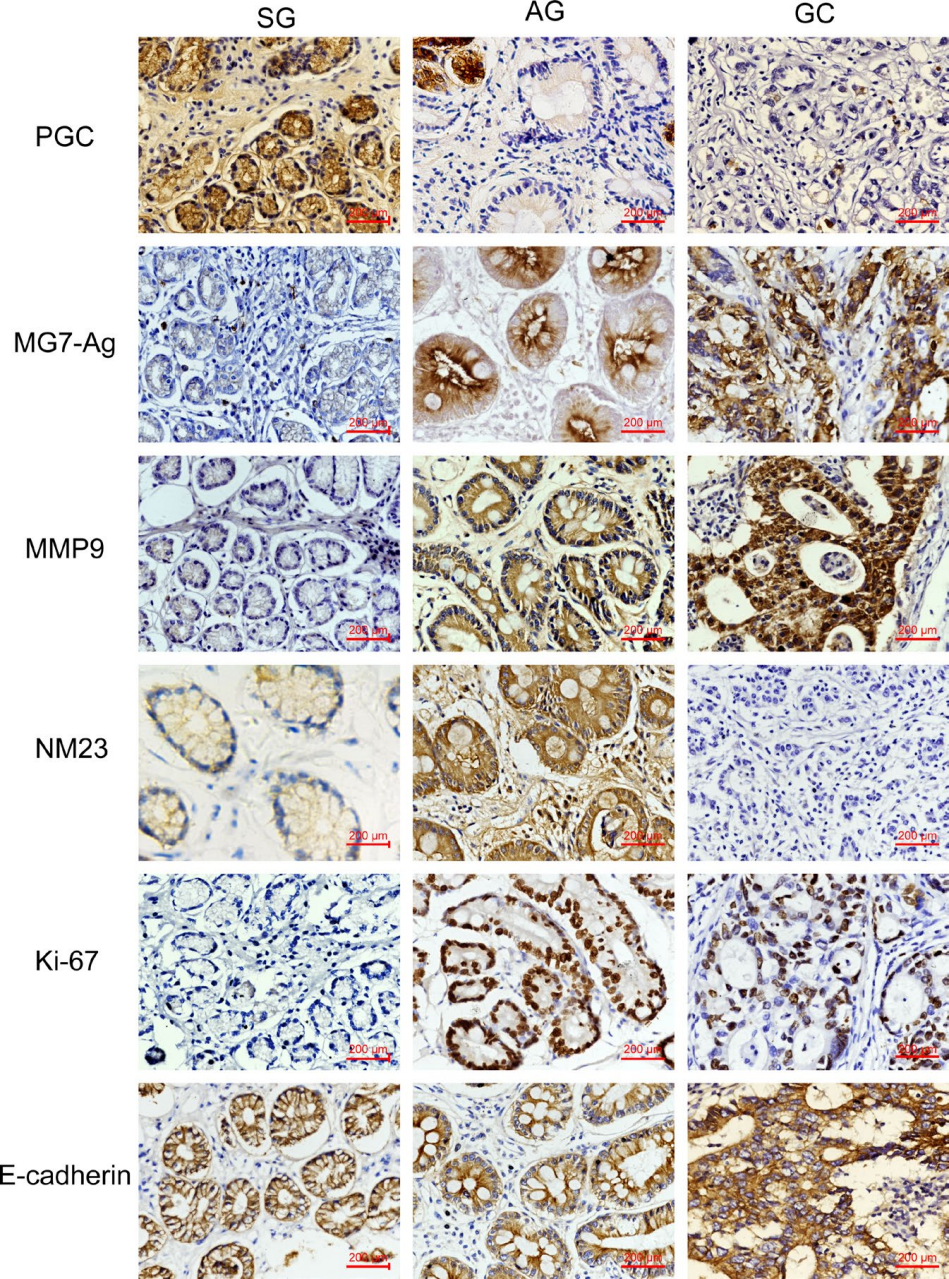
Representative photomicrographs of immunostaining of PGC, MG7‐Ag, MMP9, NM23, Ki‐67, and ECadherin in different gastric diseases specimens. Original magnification, ×400

The expression of a series of molecules related to differentiation (MG7‐Ag), migration (MMP9, NM23), cell proliferation (Ki‐67), and epithelial mesenchymal transition (E‐Cadherin) were observed in different gastric diseases. Our immunostaining results showed that MG7‐Ag expression in the GC group was higher than that of the AG and SG groups (*P* < .01), and was significantly higher in the AG group than that in the SG group (*P* < .01; Figures 2 and 3). At the same time, the expression of MMP9 was higher in the AG and GC groups compared with the SG group (*P* < .01), and significantly higher in the AG group than that in the GC group (*P* < .01; Figures 2 and 3). We also found that NM23 expression was lower in the GC group than that of the AG and SG groups (*P* < .01), and was significantly higher in the AG group than that in the SG group (*P* < .01; Figures 2 and 3). In addition, the expression of Ki‐67 in the AG and GC groups was higher in comparison with the SG group (*P* < .01). However, there was no obvious difference between the AG and GC groups (*P* = .621; Figures 2 and 3). Besides, the expression of E‐Cadherin was lower in the GC group than that of the AG and SG groups (*P* < .01), while there was no significant difference between the AG and SG groups (*P* = 1.000; Figures 2 and 3). Additionally, we analyzed the associations between the above proteins and clinicopathological parameters in the GC group (Tables [Link], [Link], [Link], [Link], [Link]‐S8).

### 3.3 Associations between PGC and tumor markers expression in different gastric diseases

**Figure 4:**
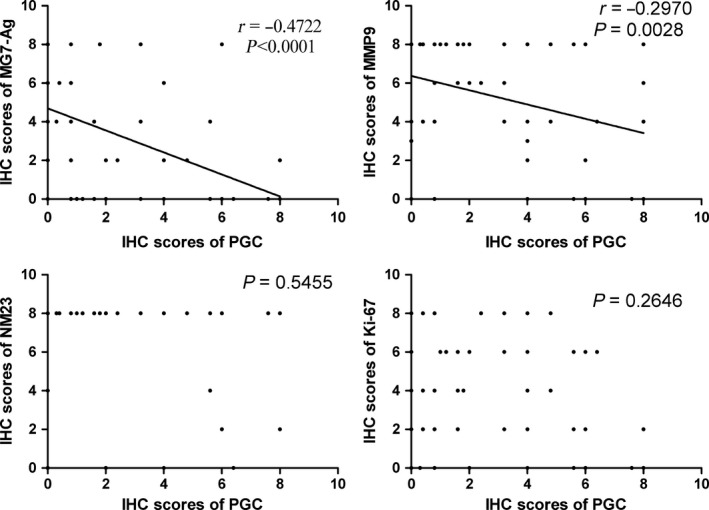
Association of PGC with MG7‐Ag, MMP9, NM23 and Ki‐67 in the AG group. Spearman's rank correlation coefficient test was adopted to analyze the correlations of different protein

In the SG group, PGC was highly expressed while the expression levels of tumor markers including MG7‐Ag, MMP9 and Ki‐67 were extremely low or lost, and there was no significant associations between them (*P* > .05; Figure S3). In the GC group, PGC was rarely expressed while the expression levels of tumor markers including MG7‐Ag, MMP9, and Ki‐67 were high, so there was also no coexpression relationship between them (Figure S4). Only in the AG group, there were different degree of expression of both PGC and tumor markers, so we analyzed their correlation. Spearman correlation analysis for immunostaining scores demonstrated that PGC expression was negatively associated with that of MG7‐Ag and MMP9 (*P* < .05), and the correlation coefficients were ‐0.472 and ‐0.297 respectively (Figure 4). However, there was no significant relationship between PGC and NM23, Ki‐67, E‐Cadherin in the AG group (*P* > .05; Figure 4).

### 3.4 Expression of PGC, MG7‐Ag, and MMP9 in cell lines

**Figure 5:**
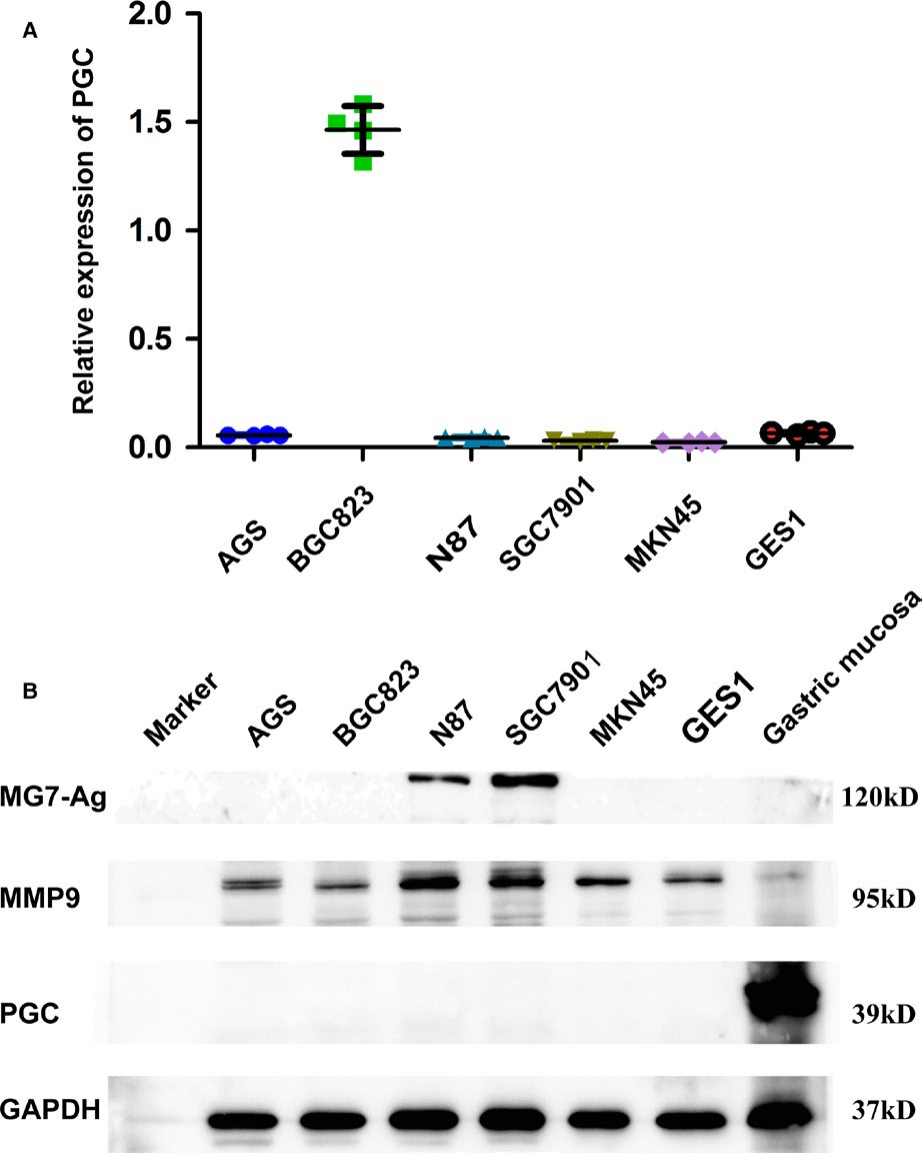
PGC expression in different gastric cancer cell lines. A, lost expression of PGC in gastric cancer cell lines were showed by qRT‐PCR. B, expression of PGC, MG7‐Ag and MMP9 were demonstrated by Western blot analysis

Furthermore, we detected the mRNA expression of PGC in gastric cancer cell lines including SGC7901, BGC823, AGS, and MKN45 and human immortalized gastric epithelial cell line (GES‐1) by quantitative real time PCR. Our results showed that PGC mRNA was undetectable in SGC7901, AGS, MKN45, and GES‐1, and only BGC823 showed low‐level expression of PGC (Figure 5A). Meanwhile we observed the expression of PGC, MG7‐Ag, and MMP9 protein by western blotting. The expression of PGC protein was not observed in all the above gastric cancer cell lines and GES1. However, considerable expression of MG7‐Ag was demonstrated in N87and SGC7901 lines and the expression of MMP9 was detected in multiple gastric cancer lines and GES‐1 (Figure 5B).

## 4 DISCUSSION

Carcinogenesis of GC is a multistep process initiated by SG and followed by AG, then IM and finally by dysplasia and GC according to the Correa's model. AG, IM, and dysplasia have been well‐established premalignant conditions of GC. Although this classical statement has been widely accepted, when and what specific kind of premalignant conditions will develop GC is still controversial. So evaluation of premalignant conditions which are most likely to turn cancerous is very important. In the present study, we determined the expression of 6 biomarkers in different gastric diseases, including differentiation (PGC, MG7‐Ag), migration (MMP9 and NM23), proliferation (Ki‐67), and epithelial mesenchymal transition‐related protein (E‐Cadherin). In addition, we analyzed the correlations between the negative marker PGC and the other positive tumor markers. The results showed that there was no coexpression of PGC and malignant phenotype markers in SG or GC tissues because of the extreme opposite expression of both. Only in AG that is well‐known to be gastric precancerous disease, coexpression of PGC and GC phenotype markers can be detected. PGC was negatively correlated with MG7‐Ag and MMP9 in AG. To the best of our knowledge, this is the first study to report the phenotypic characteristics of different stages including premalignant conditions in the development of GC from 2 aspects in combination with extremely opposite markers. This study will provide an experimental and theoretical clue to synchronously understand the molecular events of overexpression or silence of oncogene or anti‐oncogene as well as the correlation of the both in carcinogenesis of GC. Furthermore, it provides a research clue for the identification of precancerous status which may be involved in potential development into cancer.

Pepsinogen C, as the precursor of pepsin C, belongs to a family of aspartic proteinases.^10^ PGC is mainly synthesized by chief cells and then secreted into the gastric lumen where it is activated and digests food proteins.^11^ Recent studies have found that PGC plays an important role in maintaining normal morphology and physiological function of gastric epithelial cells.^22^ Our immunohistochemical results showed that PGC gradually decreased from SG, AG finally to GC. In line with our previous findings,^12^ PGC is rarely expressed in GC tissues with only about 1.4% positive rate. Furthermore, our western blot results confirmed that the expression of PGC significantly decreased and even absent in GC tissues; the same tendency was found in different gastric cancer cell lines by qPCR and western blot. The above results suggested that PGC is a weathervane of GC occurrence and development, which negatively associates with progression of gastric malignancy. Thus, PGC might be a promising negative GC marker.

MG7‐Ag is a kind of GC‐specific tumor‐associated antigen, which increases in GC and precancerous lesions.^23^, ^24^ Recent studies have suggested that MG7‐Ag could be considered an important early warning molecule of gastric cancer.^25^ MMP9, also termed as Gelatinases B, belongs to the matrix metalloproteinases (MMPs) family. MMPs, a large family of calcium‐dependent zinc‐containing endopeptidases excreted as zymogens by a variety of cells including fibroblasts, macrophages, and neutrophils, play a critical role in tissue remodeling and degradation of extracellular matrix components.^26^ Previous studies have shown that MMP9 was closely correlated with inflammation, including Helicobacter pylori‐associated gastritis and encephalitis virus.^27^, ^28^ NM23, a metastasis suppressor protein, is associated with tumor metastasis, while its expression and prognostic value in GC is still controversial.^29^ As a common marker for proliferation of tumor cells, Ki‐67 is mainly synthesized in the initial stages of cell proliferation and is present during all active phases of the cell cycle (G1, S, G2, and mitosis), but is absent in quiescent cells (G0).^30^ E‐cadherin is a Ca^2+^‐dependent transmembrane glycoprotein, which has influence on cell‐cell adhesion and the suppression of E‐cadherin function and/or expression is closely related to an EMT phenotype.^31^ E‐Cadherin is also involved in modulating cell proliferation, survival, invasion, and migration and dysregulation of which leads to dysfunction of gastric epithelial cells and promotes the development of GC.^32^ Generally speaking, the above malignant phenotype markers might be a promising positive GC marker.

Superficial gastritis is confined in the upper, foveolar portion of the mucosa beginning just below the surface epithelium,^18^ which is characterized by degeneration of gastric mucosa epithelial cells, foveolar hyperplasia and infiltration of inflammatory cells in lamina propria. AG has been defined as loss of gastric glandular cells and their eventual replacement by intestinal and fibrous tissues,^33^ frequently accompanied by intestinal metaplasia (IM) and dysplasia. SG and AG have significant differences in biological behavior and GC risk. This study revealed that the relationship between negative GC marker PGC and the above positive malignant phenotype markers is diverse in mucosa of different gastric diseases. In SG, PGC is highly expressed while malignant phenotype markers are rarely expressed and in GC, malignant phenotype markers are highly expressed while only about 1.44% PGC positive expressed, so there was not coexpression of PGC and malignant phenotype markers in both SG and GC tissues. Only in AG, coexpression of PGC and malignant phenotype markers can be found. Our results suggested that the expression of PGC in AG was negatively correlated with the expression of MG7‐Ag and MMP9, but showed no noticeable relation to NM23, Ki‐67, and E‐Cadherin. It has been estimated that 0%‐1.8%, 0%‐10%, and 0%‐73% of the patients with AG, IM, and dysplasia, respectively, will develop GC each year.^34^ Carcinogenesis from atrophic mucosa is a process from quantitative change to qualitative change. Along with greater extent and higher degree of gastric mucosal atrophy, and also with the variation in biological characteristics, the risk of developing stomach cancer will increase gradually. Recently several studies have showed premalignant conditions including AG and IM may be regressed by appropriate interventional treatment before it reaches “point of no return”,^35^, ^36^ which can reduce the incidence of GC. Therefore, precise evaluation focusing on severity and biological behaviors and proper intervention of high‐risk AG patients is of great significance for the prevention of gastric cancer. Consistent with previous studies, the positive rate of MG7‐Ag was 61.61% in the AG group.^24^ Additionally, the expression of MG7‐Ag was negatively associated with that of PGC in the AG group. Besides, our results showed that the expression of MMP9 significantly increased in AG while PGC expression significantly reduced, suggesting the expression of MMP9 in AG was negatively associated with PGC expression. Based on the above findings, we assumed that AG with low expression of PGC and high expression of MG7‐Ag and MMP9 may possess a greater potential of malignant transformation. For AG patients with high risk of GC, intervention therapy and regular follow‐up using the combined panel of PGC, MG7‐ Ag and MMP9 may be of importance for the prevention and early detection of GC.

It should be pointed out that our study is a retrospective and single‐institutional study design, carrying some inherent limitations such as selection bias. In addition, adjacent normal tissue samples used for western blot were from patients diagnosed with GC, so we cannot exclude the possibility that adjacent malignancy affects expression of proteins in the adjacent normal tissue. Further large‐scale cross‐institutional prospective studies are necessary to validate our results.

In conclusion, the expression of PGC, a marker of gastric mucosal differentiation, gradually decreased in the progression of GC and the expression of PGC was negatively correlated with that of malignant phenotype markers MG7‐Ag and MMP9. Low PGC combined with high MG7‐Ag and MMP9 may be key molecular events during malignant transformation of gastric mucosa. The promising combination of negative marker PGC and positive markers MG7‐ Ag and MMP9 could be used as a potential follow‐up panel for monitoring dynamical progression of AG and to improve the detection efficiency of high‐risk individuals of GC, so then to take the necessary interventions on the target population.

## CONFLICT OF INTEREST

The authors declared that there are no competing financial interests.
