# Supplementary material for: Correlation between negative expression of pepsinogen C and a series of phenotypic markers of gastric cancer in different gastric diseases
Source: Cancer Med. 2018 Jul 2;7(8):4068–76. doi: 10.1002/cam4.1615 (PMC6089156; doi:10.1002/cam4.1615)
Supplement: Supplementary file 1 [file CAM4-7-4068-s001.docx]

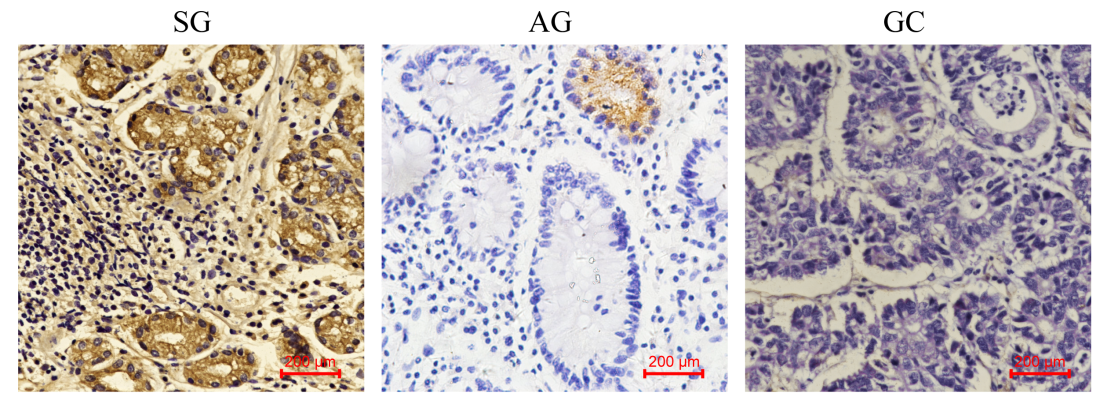


Supplementary Figure S1. Representative immunostaining of PGC in specimens of different gastric diseases. The expression levels of PGC in AG and GC were higher than in SG. Original magnification, ×400.


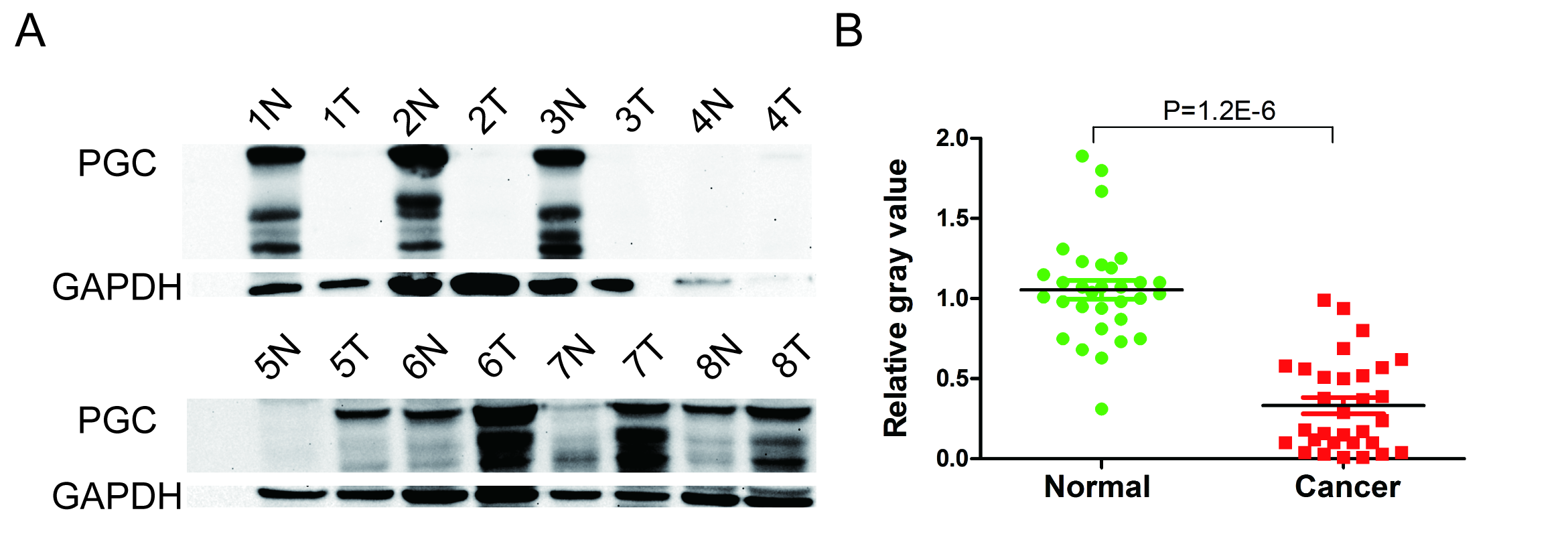


Supplementary Figure S2. Western blot analysis of PGC expression in 31 pairs of gastric cancer and adjacent normal tissues. A. Representative immunoblot images of PGC are shown. N: adjacent normal tissues; T: gastric cancer tissues. B. densitometry analysis of western blot, the expression of PGC was qualified compared with the expression of GAPDH. The differences of gray values of PGC/GAPDH between gastric cancer tissues and adjacent normal tissues was analyzed by Wilcoxon Signed Ranks.





Supplementary figure S3. Association of PGC with MG7-Ag, MMP9, NM23 and Ki-67 in SG. The correlation of different protein was measured by Spearman's rank correlation coefficient test.

Supplementary figure S4. Association of PGC with MG7-Ag, MMP9, NM23 and Ki-67 in GC. The correlation of different protein was measured by Spearman's rank correlation coefficient test.


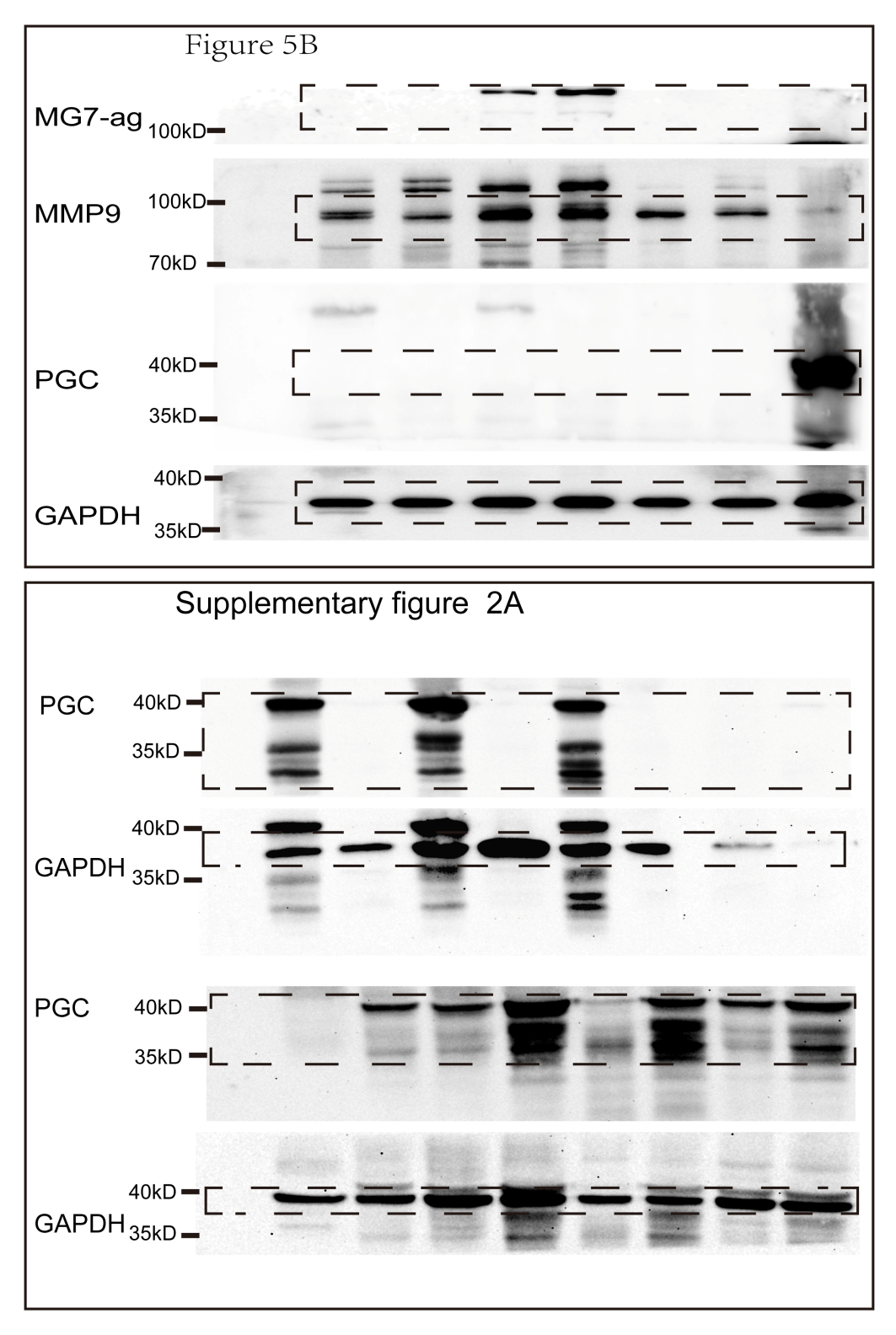


Supplementary Figure S5. Original blots from figures. Dashed box indicates cropped images shown in the corresponding figures.
